# A Novel Long Non-coding RNA, *durga* Modulates Dendrite Density and Expression of *kalirin* in Zebrafish

**DOI:** 10.3389/fnmol.2017.00095

**Published:** 2017-04-10

**Authors:** Mayuresh A. Sarangdhar, Divya Chaubey, Abhishek Bhatt, Monisha KM, Manish Kumar, Shashi Ranjan, Beena Pillai

**Affiliations:** ^1^Functional Genomics, Council of Scientific and Industrial Research (CSIR), Institute of Genomics and Integrative Biology (IGIB)New Delhi, India; ^2^Academy of Scientific and Innovative Research (AcSIR)New Delhi, India

**Keywords:** Kalirin, long non-coding RNA, dendritic morphology, zebrafish, primary neuron culture

## Abstract

Kalirin, a key player in axonal development, nerve growth and synaptic re-modeling, is implicated in many pathological conditions like schizophrenia and autism-spectrum disorders. Alternative promoters and splicing lead to functionally distinct isoforms, but the post-transcriptional regulation of Kalirin has not been studied. Here, we report a novel non-coding RNA, which we name *durga*, arising from the first exon of kalirin a (*kalrna*) in the antisense orientation in zebrafish. The *kalrna* and *durga* transcripts are barely detectable during early development, but steadily increase by 24 hours post-fertilization (hpf) as the brain develops. Over-expression of *durga* in the zebrafish embryo led to an increase in *kalrna* expression. The morphology of the neurons cultured from *durga* injected embryos had significantly fewer and shorter dendrites. Although *durga* has no apparent sequence homolog in mammals, based on gene synteny, we found a non-coding RNA arising from the 5′ end of the human *Kalrn* gene and expressed in the human neuronal cell line, SH-SY5Y. We propose that the zebrafish lncRNA *durga* maintains dendritic length and density through regulation of *kalrna* expression and this may have further implications in mammalian systems.

## Introduction

Development of the brain involves a fine balance between excitatory and inhibitory signaling at synapses, ensuring plasticity while avoiding excitotoxicity. Kalirin, originally reported as a protein that interacts with Huntington associated protein (HAP), is known to be involved in active remodeling of synapses (Colomer et al., 1997). Kalirin has been implicated in a variety of neuropsychiatric and neurodegenerative diseases like schizophrenia, depression and Alzheimer’s disease (Hill et al., 2006; Mandela and Ma, 2012; Remmers et al., 2014; Makrythanasis et al., 2016). At the molecular level, this RhoGEF kinase is expressed highly at excitatory synapses and reduced at inhibitory synapses. Its binding to receptors leads to RhoGTPase mediated signaling and regulation of actin cytoskeletal organization. This intra-cellular signaling pathway leads to synaptic remodeling through altered dendrite numbers and dendritic morphology (Penzes et al., 2001). The mammalian Kalirin (*Kalrn*) gene locus gives rise to several isoforms which have diverse and sometimes antagonistic effects on dendritogenesis. The *Kalrn* gene can code for a protein with a lipid binding motif, several tandem spectrin homology domains and additional protein-protein interaction domains like SH3 domain commonly found in members of signaling pathways (McPherson et al., 2002, 2004; Vishwanatha et al., 2012; Miller et al., 2017). The functional differences between isoforms are thought to arise from the combinations of protein domains retained in the splice isoforms. The effects of alternative isoforms of *Kalrn* on dendritic morphology have been studied extensively (Penzes et al., 2001; McPherson et al., 2002, 2004; Vishwanatha et al., 2012). However, the regulation of *Kalrn* transcription during development has not been explored extensively.

The human *Kalrn* gene is located on chromosome 3 and spans a huge 0.63 Mb region with a complex gene structure encompassing 17 alternatively spliced transcripts. The largest transcript of 19.9 kb contains 60 exons (McPherson et al., 2002). The shorter isoforms of human *Kalrn* fall into two broad categories, the ones containing combinations of the 30 exons from the 5′ end or 20 exons from the 3′ end. Johnson et al. (2000) and McPherson et al. (2002) reported the domain architecture of Kalirin protein isoforms and their relation to the alternative spliced transcript isoforms. *Kalrn7*, the major brain-specific isoform, and the closely related *Kalrn8* isoform consist of the core Sec14p-like putative lipid-binding motif, nine spectrin-like repeats and a Dbl homology/pleckstrin homology (DH-PH) domain but differs at the amino terminus and have a distinct PDZ binding motif or an SH3 motif at the -COOH end respectively. *Kalrn9* and *Kalrn12* have additional C-terminal domains and show distinct sub-cellular localization in cortical neurons while *Kalrn7* shows characteristic punctate localization in the neuronal processes. The transcript isoforms also have distinct spatio-temporal expression patterns with *Kalrn7* being expressed in the adult brain, while *Kalrn9* and *Kalrn12* are expressed in the developing brain and yet another isoform Trio is expressed in many tissues (Hansel et al., 2001).

Zebrafish is emerged as a convenient model to explore axonal growth (Chen et al., 2013), signaling by neurotropic factors during development and in human diseases. Thus, we studied the *kalrna* locus in detail on chromosome 9 of the zebrafish genome (Zv10). The 2.9 kb predicted transcript from the longest isoform (ENSDART00000164543.1) consists of the expected core domains (spectrin repeats, DH-PH domains). The *kalrna* locus has been extensively re-annotated during the transition from zebrafish genome version 9 (Zv9) to version 10 (Zv10). A notable change is the extension of the 5′ end to include an exon positioned 95 kb upstream to the previously reported *kalrna* gene. We noticed that in Zv9, this region was inverted and hence the link with the *kalrna* gene was not evident. Large scale studies, most notably, the ENCODE project, have shown that extended 5′ terminal exons can play an important role in the regulation of the gene. On closer inspection, we found that the region also corresponds to an lncRNA reported by Kaushik et al. (2013) from an RNA-seq based developmental profiling of non-coding RNAs.

Here, we show that this lncRNA is transcribed in anti-sense to the largest *kalrna* transcript in zebrafish. The multi-domain Kalirin was named after the multi-armed goddess Kali from Indian mythology (Alam et al., 1997), and hence, we propose to name this lncRNA that is anti-sense to *kalrna*, *durga*- the mythological alter-ego of Kali. The lncRNA is expressed in the oocyte and as embryo is developing, it gets gradually restricted to the brain. The presence of this lncRNA seems to be critical for appropriate temporal expression of *kalrna*. The *durga* transcripts present in the cytoplasm of the fertilized embryo, subsequently increases and localize to the developing zebrafish brain. The rise in *durga* expression is accompanied by an increase in the level of *kalrna* mRNA and coincides with the developmental time when the zebrafish brain is being formed and neurites are growing as rapidly as 20 microns/h (Chen et al., 2013). Ectopic injection of the *durga* RNA results in further increase in the expression of *kalrna*. Primary zebrafish neurons with elevated *durga* and *kalrna* showed striking morphological changes in dendrites. Using a transgenic fish with neurons marked with *kaede*, we found that the ectopically injected lncRNA triggered a profound loss of dendrites, perhaps corresponding to the increased *kalrna* expression. Thus, transcription from the first exon of the *kalrna* gene, located about 95 kb upstream to the next exon in zebrafish, seems to be regulated by lncRNA *durga*.

## Materials and Methods

### Animal Husbandry

Zebrafish (*Danio rerio*) stocks were maintained according to standard procedures (Westerfield, 2000) with 13/11 h light/dark cycle at the zebrafish facility at Institute of Genomics and Integrative Biology (IGIB). Zebrafish experiments were performed in strict accordance with the recommendations and guidelines of the CSIR-Institute of Genomics and Integrative Biology, India. The protocol was approved by the Institutional Animal Ethics Committee (IAEC) of the CSIR-Institute of Genomics and Integrative Biology, India. Embryos from *casper* (White et al., 2008) and [Tg(*HuC*: *Kaede*)] (Sato et al., 2006) strains were used for all experiments. Fish were paired in mating cages a night before obtaining embryos. Embryos were collected and harvested at different stages of development and eggs were obtained by squeezing abdomen of gravid females.

### LncRNA *durga* Over-Expression by Microinjections

LncRNA *durga* was over-expressed at one-cell stage by injecting 5 ng of *in vitro* synthesized (MEGAshortscript transcription kit, Thermo Fisher Scientific) RNA. The embryos were monitored constantly and fixed at 1cell, high (3 hpf), shield (6 hpf), 2Somites (11 hpf) and 30Somites (24 hpf) for *in situ* hybridization and RNA isolation.

### RNA Extraction, Reverse Transcriptase and Polymerase Chain Reaction

Total RNA was extracted using TRIzol (Invitrogen) as per manufacturer’s instructions from mentioned time points. cDNA was synthesized using 500 ng total RNA with 5000 pg spiked in exogenous RNA with gene specific reverse primer or random hexamer primers and M-MLV reverse transcriptase at 42°C for 1 h. Standard polymerase chain reaction (PCR) was performed to amplify *durga* and *kalrna* with intron spanning primers.

### Real Time Polymerase Chain Reaction

Real-time PCR (RT-PCR) for *durga* and *kalrna* was performed using spiked in exogenous RNA as internal control. SYBR green master mix (Takara) was used as per manufacturer’s protocol on Roche LightCycler480. Data was extracted and analyzed manually using excel.

### RNA Detection by *In Situ* Hybridization

lncRNA *durga* and *kalrna* specific sequence was amplified using specific primers from adult brain cDNA and cloned in TOPO2 dual promoter vector (Invitrogen). Plasmids were linearized using SpeI and ECoRV restriction enzymes and probes were *in vitro* synthesized using T7 and SP6 RNA polymerase and digoxigenin labeled UTPs. Embryos were fixed using 4%w/v PFA(Sigma) made in 1× PBS, pH 7.4. Embryos were washed with 1× PBS 0.1%, tween and stored at −20°C in 100% methanol. Embryos were hybridized with lncRNA *durga* and *kalrna* antisense probes in hybridization buffer at 65°C overnight, thereafter given stringency washes with 25%, 50%, 75% and 100% 2× SSC. Embryo were then washed with maleic acid buffer with 0.1% tween. Embryos were then incubated with digoxigenin-alkaline phosphatase Fab fragments (Roche) in 1:1000 dilutions overnight at 4°C. Post antibody incubation, stainings were developed using Nitro-blue tetrazolium (NBT) and 5-bromo-4-chloro-3′-indolyphosphate (BCIP) substrate (Roche). After completion of staining, reaction was terminated by fixing embryos with PFA. Embryos were then taken serially through 25%, 50% and 75% of glycerol and imaged in 2.5% methyl cellulose.

### Northern Blotting

Total RNA was extracted from zebrafish adult brain and 30 μg of total RNA was run on denaturing agarose gel. RNA was then transferred to Amersham hybond N^+^ membrane by capillary action and then crosslinked to the membrane using stratalinker. Probes for lncRNA and *kalrna* were made using 50 μCi αP^32^ dATP and Klenow polymerase (M0210S, NEB) incubating reaction mix at 37°C for 1 h. Blot was prehybridized in Church buffer for 3 h at 65°C and incubated in hybridization buffer overnight. Blot was then washed with 2× SSC + 0.1% SDS, 1× SSC + 0.5% SDS and 0.1× SSC + 0.5% SDS serially for 1 h each at 65°C with agitation. Blot was then exposed to the film for 45 h and film was scanned in phosphoimager to obtain an image.

### Zebrafish Primary Neuron Culture

Control or *durga* over-expressed 48 hpf embryos were decorionated and deyolked under microscope. Then they were washed with sterile cold PBS in cell culture hood. Embryos were disintegrated by adding Trypsin and incubating at 37°C for 20 min, with intermittent mixing. Trypsin was removed by centrifugation. Fresh Neurobasal-A media was added to trypsinized embryos and triturated slowly and carefully with pipette to get single cell suspension (Westerfield, 2000). Cells were passed through 70 μm mesh to get rid of clumps and plated in poly-D lysine coated plates or coverslip in Neurobasal-A medium with B27, glutamax and primocin. Cultured cells were incubated at 29°C in the CO_2_ incubator for 24 h, then collected for RNA isolation or fixed for imaging.

### Imaging Acquisition and Analysis

Images of *in situ* hybridized embryos were captured using NIKON SMZ800N microscope at 6× magnification. Fluorescence images of primary cultured neurons were captured using LeicaTCSSP8 microscope at 100× magnification using 488 laser in randomly selected fields. Dendritic density and length was measured using NeuronJ plug-in of the ImageJ tool in the images of control or *durga* over-expressed neurons (Meijering et al., 2004).

## Results

The advent of next generation sequencing technologies has led to the rapid, high-throughput identification of thousands of putative non-coding RNAs. The zebrafish genome (latest version Zv10) has been recently re-annotated to incorporate modifications at many loci. We set out to study a putative lncRNA locus that was re-annotated in the recent version of the zebrafish genome.

### Genomic Organization of Zebrafish *kalrna* in Zv9 and Zv10 Genome Assembly

**Figure 1:**
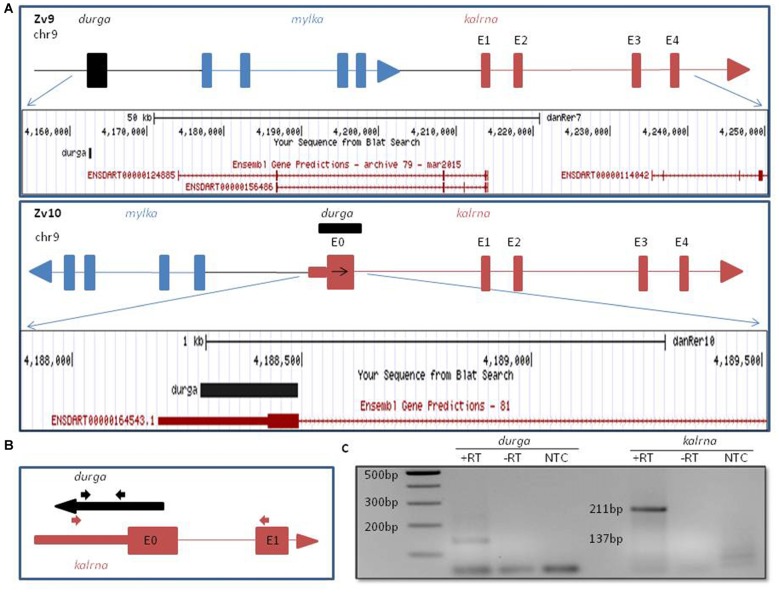
**Genomic organization of zebrafish *kalrn* in zebrafish genome version 9 (Zv9) and zebrafish genome version 10 (Zv10) genome assembly. (A)** Pictorial representation and UCSC genome browser tracks of gene re-arrangement of *kalrna*, *mylka* and *durga* in the Zv9 and Zv10 genome assembly. **(B)** Primers are shown with their position and orientation. Representation not to scale. **(C)** Expression of *durga* (137 bp) and novel first exon of *kalrna* (211 bp) was confirmed by polymerase chain reaction (PCR) with cDNA synthesized from strand specific primer and zebrafish brain RNA.

In the Zv9 version of the zebrafish genome, *mylk1* and *kalrna* were assembled in a convergent orientation on the same strand with ~20 kb intergenic region. However in Zv10, this was revised to include a genomic inversion, which places *mylk1* and *kalrna* in the divergent orientation on opposite strand, separated by ~11 kb intergenic region (Kent et al., 2002; Figure 1A). Apart from changes in the orientation, we also noticed the addition of a poorly conserved 5′ exon (denoted as E0 in Figures 1A,B), ~95 kb upstream to the previously reported *kalrna* transcript which confers a 22 amino acids N-terminal extension. Interestingly, this 5′ exon also includes a 240 nucleotide long 5′UTR, which was not annotated in Zv9. Such extended or alternative 5′ terminal exon can play an important role in the regulation of the mRNA stability and translation by interacting with translational machinery, microRNAs or lncRNAs. Regulation of *kalrna* by lncRNA is not known. Thus, we checked for presence of any lncRNA near the 5′ locus of *kalrna* in the zebrafish lncRNA database zflncRNApedia (Dhiman et al., 2015). We found one such lncRNA ZF_LNC002146 in the database; although its location is ~72 kb away from *kalrna* according to Zv9 annotation, overlaps with extended 5′ exon of *kalrna* in Zv10 (Figure 1A). This lncRNA was first reported as lncBR47, to be highly enriched in the brain (Kaushik et al., 2013). However, in this study, lncRNAs were detected using a RNA-seq data generated by non-directional RNA-seq method, suggesting that the lncBR47 (named *durga* hereafter) may correspond to the *kalrna* first exon. Therefore, it was not clear if the reported lncRNA is in fact part of an extended isoform of *kalrna* or a distinct lncRNA antisense to *kalrna*.

To verify which of the two strands are transcribed at the *kalrna* locus, we used gene specific reverse primers to synthesize cDNA from zebrafish brain RNA (Figure 1B; Table 1). Both the cDNA samples gave rise to expected size PCR products suggesting that the locus is transcribed bi-directionally (Figure 1C). We further confirmed the expression of both transcripts by northern blotting and oligodT primed RT-PCR (Supplementary Figure S1).The positive strand would produce a *kalrna* isoform while the negative strand would give rise to *durga*.

**Table 1:** **Primer sequences used**.

No.	Primer name	Primer sequence	Used in Figure
1	*durga*_F.P 1	CCTCTTGTATCTCACAGCTCAA	Figure 1C
2	*durga*_R.P 1	AGACAATAGAAGGCAATGCAG	Figure 1C
3	*kalrna*_F.P 1	CTGGAGACAATAGAAGGCAAT	Figure 1C
4	*kalrna*_R.P 1	GGAAGGACATCAGAGGCTTTAATTC	Figure 1C
5	*durga*_F.P 2	CGCTCCATCATCTCAGTGTG	Figures 2A,B, 3A, 4A
6	*durga*_R.P 2	AGACAATAGAAGGCAATGCAG	Figures 2A,B, 3A, 4A
7	*kalrna*_F.P 2	CTGAGATCTGCGTCGCTCTCATC	Figures 2D,E, 3C, 4A
8	*kalrna*_R.P 2	CAAAGTCGTCCGTTAGCTGGG	Figures 2D,E, 3C, 4A
9	Hu_*kalrn*_nc_FP	AGTGTGAAGGTGTGGGAGTTG	Figure 5B
10	Hu_*kalrn*_nc_RP	TGCATTCAGTCATCCTTGTCTC	Figure 5B

*durga nucleotide sequence: GTTTTGAAGAATGCATCCGTGTCCGAATCATTGGGTCCGTCCTCCGGCGCTTCAGCAGAATTCATGTTTTCCTCTTGTATCTCACAGCTCAAAATAACAAGAAATATATCCAAATTCAGCGCTCCATCATCTCAGTGTGTCTGTTTTTGACGGCACAGCCCTCAGTCAGCGCGACAAAGCACAGCTCTGCATTGCCTTCTATTGTCTCCAG*.

### Developmental Expression Profile of *durga* and *kalrna* in Zebrafish

**Figure 2:**
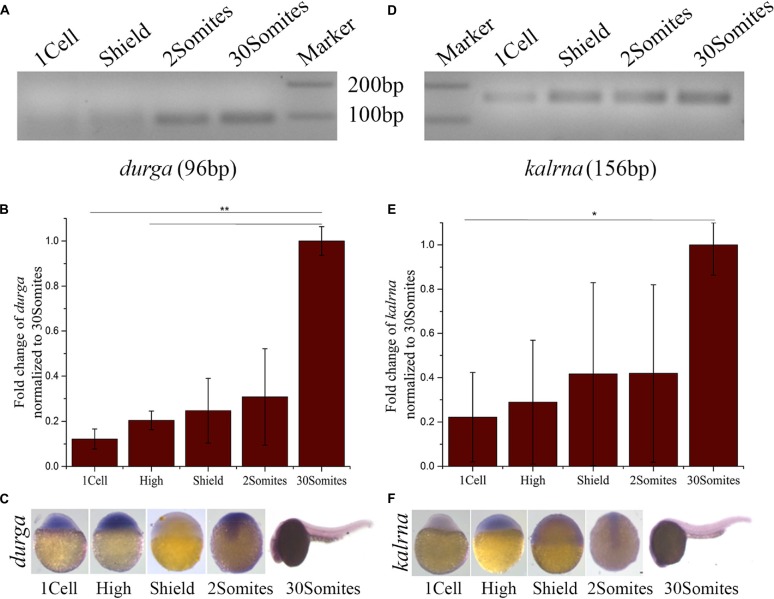
**Developmental expression profile of *durga* and *kalrna* in zebrafish. (A)** Expression of *durga* and *kalrna* was checked by semi-quantitative PCR (**A,D** respectively), qPCR (**B,E** respectively) and *in situ* hybridization (**C,F** respectively) during 1cell, High, Shield, 2Somites, 30Somites stages. Data shown represent more than three independent experiments. *p*-values were calculated using Student’s *t*-test. **p ≤ 0.01, *p ≤ 0.05.

To study the spatio-temporal expression pattern of *durga*, we used gene-specific reverse primers for cDNA synthesis and primer pairs were designed to produce PCR products of different sizes corresponding to *kalrna* and *durga* (Table 1). The reverse transcriptase-PCR analysis showed that *durga* (Figures 2A,B; 96 bp) is expressed at low levels during the early stages of development but steadily increases by 24 hpf (30Somites). We also used riboprobes from the sense (with respect to *kalrna*) and anti-sense strand in *in situ* hybridizations to confirm that both strands produce transcripts *in vivo*. *In situ* hybridization confirms the presence of *durga* transcripts in the egg (Supplementary Figure S2) and a clear expression throughout the developing embryo upto 11 hpf (2Somites stage). Subsequently as the brain develops, the expression of the lncRNA becomes localized to the head region of the embryo (Figure 2C). The *kalrna* starts expressing after zygotic genome activation and becomes apparent in anterior region of the embryo as brain starts developing at 2Somites stage (Figures 2D–F; 156 bp). By 24 hpf, the *kalrna* expression is almost entirely in the anterior region (Figure 2F).

### LncRNA *durga* Modulates the Expression Profile of *kalrna*

**Figure 3:**
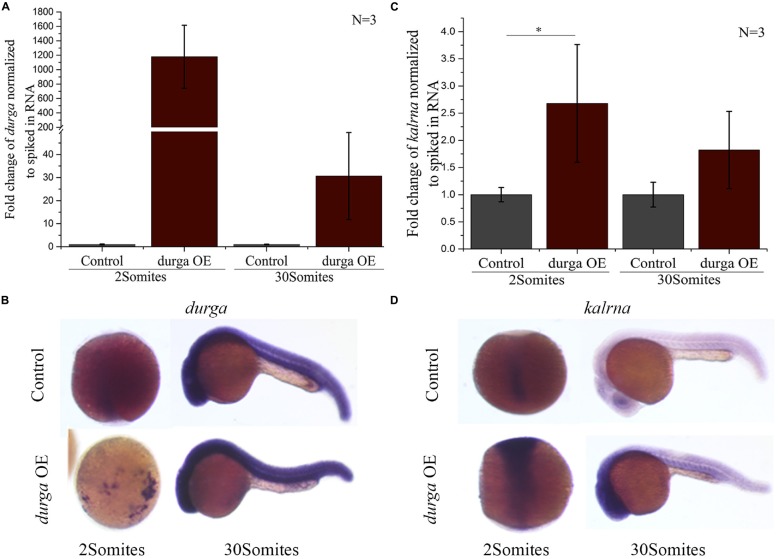
**Over-expression of *durga* enhances *kalrna* expression.** *In vitro* transcribed *durga* was injected in one cell stage of zebrafish embryo and over-expression was confirmed by qPCR **(A)** and *in situ* hybridization **(B)** at 2Somites and 30Somites stages. Increase in *kalrna* expression was seen in qPCR **(C)** and *in situ* hybridization **(D)** at 2Somites and 30Somites stages. Data represent three independent experiments. *p*-value was calculated using Student’s *t*-test. *p ≤ 0.05. OE denotes overexpression. *N* is number of biological replicates.

Like *kalrna* and *durga* many lncRNAs are found in close proximity of the genes they regulate and share spatio-temporal expression patterns with their targets (Yap et al., 2010; Stojic et al., 2016; Tran et al., 2016). To find if *durga* could affect the expression profile of *kalrna*, we injected *in vitro* transcribed *durga* RNA into the embryo at the 1-cell stage. The injected RNA was stable upto the 30Somites stage and localized to the anterior region, matching the spatial expression pattern of the endogenous *durga* transcripts (Figures 3A,B). In these embryos, *kalrna* showed a two-fold upregulation in quantitative RT-PCR (Figure 3C). A strong upregulation of *kalrna* was also evident in the brain region in *in situ* hybridization experiments (Figure 3D). The induction of *kalrna* is more evident in *in situ* hybridization perhaps because of the dilution due to homogenization of the whole embryo in the quantitative RT-PCR.

### LncRNA *durga* Alters Dendritic Morphology in Primary Culture of Zebrafish Neurons

**Figure 4:**
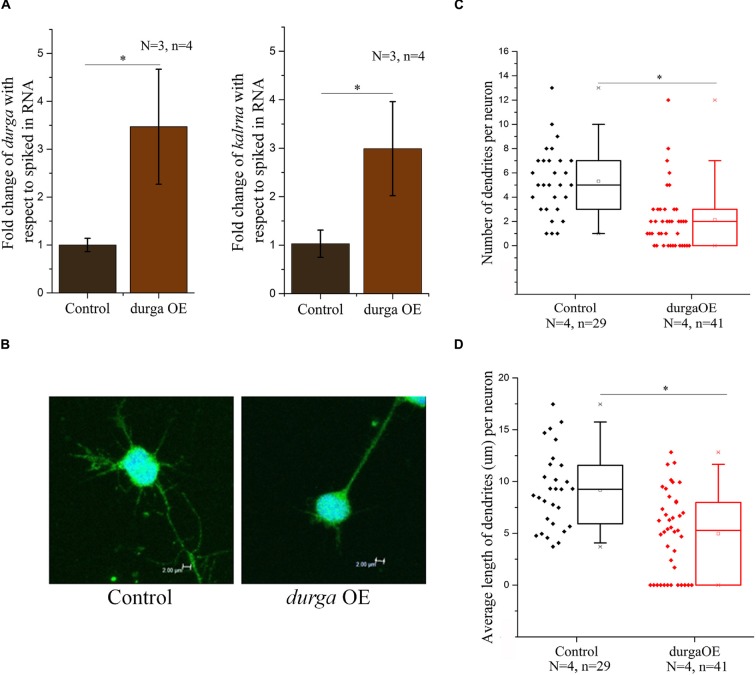
**Over-expression of *durga* in zebrafish embryos alters dendritic morphology in primary culture of neurons.** Over-expression of *durga* and *kalrna* was confirmed by qPCR in primary culture of neurons **(A)**. Confocal image of *durga* over-expressed neuron showed reduced average dendrite number **(B,C)** and dendrite length **(B,D)**. Data shown represent minimum three independent experiments. *p*-values were calculated using Student’s *t*-test. *p < 0.05. OE denotes overexpression.

*kalrna* is a guanine nucleotide exchange factor (GEF) that affects cytoskeletal arrangement in neurons, resulting in changes in dendritic spine morphology. During development, spine dynamics plays an important role in the proper formation of neuronal circuits, while in adults spine morphogenesis is linked to synaptic plasticity, learning and cognition. By virtue of its role in neuritogenesis and dendritic spine morphology, *kalrna* is implicated in several neuro-psychiatric diseases like schizophrenia, Alzheimer’s disease and depression. Therefore, we were interested in testing the effect of the lncRNA *durga* on neuronal cell morphology. We used a double transgenic zebrafish line with radial glial cells marked with mCherry driven by a *Her4.1* promoter (Yeo et al., 2007) and neuronal cells marked by the *kaede* reporter under the regulation of the *HuC* promoter (Sato et al., 2006). After 48 hpf, *durga* injected and control embryos were triturated and plated in Neurobasal-A medium to study the morphology of individual neurons. In these cells, we confirmed that the over-expression of *durga* and up-regulation of *kalrna* were consistent with the effects we found *in vivo* (Figure 4A). A closer examination of the morphology of the neurons showed that cells from *durga* injected cells had significantly fewer dendrites (Figure 4B). As shown in Figure 4C, 60% of the control cells showed more than five dendrites per cell while 75% of the *durga—kalrna* over-expressing cells had less than five dendrites per cell (Figure 4C). The average length of dendrites also reduced significantly in the *durga—kalrna* over-expressing cells (Figure 4D).

## Discussion

Kalirin acts a pivotal point in the regulation of cytoskeletal organization and thus neuronal morphology because of integration of diverse, excitatory as well as inhibitory signals. Human *Kalrn* gene is a large gene that shows extensive alternative splicing leading to multi-domain protein isoforms whose functionality is determined by the combination of exons included in each transcript type. The alternative transcripts are of two broad categories, the ones that have predominantly 5′ exons, for instance, *Kalrn*-7, 9, 12 and others that have predominantly 3′ exons like Duo and Trio. These transcript isoforms also differs in the spatio-temporal expression pattern as seen in *Kalrn7* found in the adult brain and *Kalrn9* and *12* predominantly expressed during development. Kalirin protein isoforms seem to respond to a variety of signals including Nerve Growth Factor signaling through the TrkB receptor, BDNF signaling through Rac1 (Chakrabarti et al., 2005; Yan et al., 2016). It also interacts with many PDZ domain containing proteins such as PSD95 (Penzes et al., 2001). Kalirin proteins can integrate these signals to modify actin cytoskeletal dynamics eventually modifying neurite morphology and axonal growth of neurons.

We noted a putative, brain enriched lncRNA that directly overlaps with the extended exon of the *kalrna* gene in the Zv10 annotation of the zebrafish genome. Using a combination of semi-quantitative RT-PCR with directional primers and *in situ* hybridization, we established that the locus gives rise to an lncRNA *durga* and the transcript of *kalrna* gene. Both *durga* and *kalrna* showed increasing expression during development, finally localizing to the brain region by 24 hpf. Further, over-expression of the lncRNA resulted in an increase in the expression of *kalrna* and decrease in the number of dendrites in zebrafish neurons. Currently, no detailed information on the transcript isoforms of *kalrn* in zebrafish is known. However in future, it will be interesting to explore the isoform-specific effects of the *durga* in neurodevelopment and neurodegenerative disorders. Besides *durga*, additional factors are likely to be involved in the regulation of zebrafish *kalrna*.

Depletion of the endogenous *durga* lncRNA is technically challenging due to the overlap with the *kalrna* gene. Standard tools like CRISPR or morpholinos are not readily applicable. CRISPR based editing would disrupt the *kalrna* gene on the opposite strand. Lack of information on exon-intron structure in *durga* gene prevented use of morpholinos to knock-down the expression of the lncRNA. In future, it will be interesting to study the mechanism of *kalrna* regulation by *durga*.

The *Kalrn* over-expression, especially in the form of the transcript isoform *Kalrn7* is usually associated with increased dendritic length while *Kalrn9* and *12* isoforms seem to have age dependent, contrasting effects on dendritic length. In early development, *Kalrn9* and *12* isoforms induce dendritic elongation while in mature neurons *Kalrn9* over-expression reduces dendritic length (Grubisha et al., 2016). The dual role of *Kalrn9* and *12* isoforms to induce or retract dendrites comes from their two GEF domains. The first GEF domain activates RhoG/Rac1, which promotes the formation and elongation of dendrites. The second GEF domain interacts with RhoA, which induces spine elimination (Tolias et al., 2011; Yan et al., 2015). The *Kalrn7* isoform does not have a second GEF domain and thus over expression of *Kalrn9* and *12* isoforms but not *Kalrn7* reduces dendritic length (Deo et al., 2012; Grubisha et al., 2016). Interestingly *Kalrn9* expression has been observed to be increased during normal human aging in the cortex and also in schizophrenia subjects (Deo et al., 2012; Grubisha et al., 2016).

Reduction in dendritic length, pruning of synaptic connections and decrease in brain size are common pathological symptoms of neurodevelopmental, neuro-degenerative and neuro-psychiatric disorders like autism, schizophrenia, Alzheimer’s (Remmers et al., 2014). On the other hand, controlled synaptic pruning and maintenance of dendritic arborization are also essential to maintain structural plasticity of brain throughout life. lncRNAs have been reported to be involved in almost every aspect of brain development with diverse mechanisms of action. For example, lncRNA POU3F2, Gomafu, HAR1F are involved in neural stem cell proliferation while lncRNA Pnky, NEAT1, TUG1, EVF2 regulates neuronal differentiation (Iyengar et al., 2014). Besides, the role of lncRNAs in regulation of neuronal architecture and synaptic plasticity is beginning to be understood. Here we showed that lncRNA *durga* shortens the dendrites and reduces dendritic arborization in zebrafish, which may have higher level implications during neurodevelopment, neuropathologies and aging.

**Figure 5:**
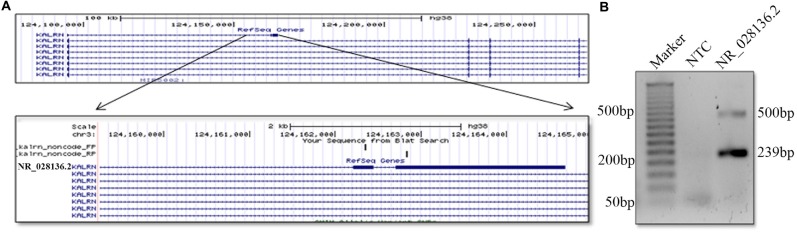
**Presence of non-coding RNA at 5′ end of human *Karln*.** Pictorial representation and UCSC genome browser tracks of non-coding (NR_028136.2) RNA located at 5′ end of human *Kalrn*. Representation is not to scale **(A)**. Expression of non-coding RNA (NR_028136.2) located at 5′ end of human *Kalrn* was confirmed by PCR in SH-SY5Y cell line **(B)**.

lncRNAs are poorly conserved at nucleotide levels, but criteria of sequence level conservation is too narrow for lncRNAs. Large number of lncRNAs shows structural, functional conservation and expression from syntenic loci despite lack of sequence homology (Diederichs, 2014; Johnsson et al., 2014). We found that the 5′ end of human and mouse *Kalrn* locus gives rise to non-coding RNA. Although the sequence of *durga*, human and mouse lncRNA is not conserved, the gene arrangement is similar (Figure 5A). Putative human counterpart of *durga* has three exons and its first exon has overlap with first exon of *Kalrn*. Further expression of human *Kalrn* non-coding RNA was checked by PCR using cDNA prepared from RNA of SH-SY5Y cell line. We found both spliced (239 bp) as well as unspliced (500 bp) form of human *Kalrn* non-coding RNA (Figure 5B).

In both the mammalian genomes, the lncRNA is annotated in the same orientation as the *Kalrn* gene, while in zebrafish it is anti-sense and overlapping. Further functional studies and evaluation of *durga* in mouse model of neurodegenerative diseases may pave a way for better understanding of *Kalrn* regulation during development and in various neuropathologies.

## Conclusion

Kalirin regulates higher level brain functions by modulating dendritic spine density and morphology. We find an antisense, promoter-proximal lncRNA co-expressed with *Kalirin* that affects dendrite length and density in zebrafish.

## Author Contributions

BP conceived the idea. BP and MAS designed the experiments. DC and MKM collected zebrafish samples. MKM and SR maintained zebrafish stocks. MAS and DC performed PCR, qPCR, primary cell culture experiments and data analysis. DC did Northern blotting experiments. AB and DC performed the *in situ* hybridization experiments. MK captured the confocal images. BP, MAS and DC co-wrote the article.

## Conflict of Interest Statement

The authors declare that the research was conducted in the absence of any commercial or financial relationships that could be construed as a potential conflict of interest.

## Supplementary Material

FIGURE S1 **Northern blot representing transcript corresponding *kalrna* and lncRNA *durga***. Northern blotting experiment shows the presence of transcripts corresponding *kalrna* and *durga* above 5 KB and ~400 bp respectively **(A)**. Polymerase chain reaction (PCR) detection of *durga* transcript using anchored oligodT reverse primer for cDNA synthesis and amplified using *durga* forward primer **(B)**.

FIGURE S2 ***In situ* hybridization of *kalrna* and *durga* in the zebrafish egg.** *In situ* hybridization shows *kalrna* and *durga* transcript expression in zebrafish egg.
