# Supplementary figures and images for: A Novel Long Non-coding RNA, durga Modulates Dendrite Density and Expression of kalirin in Zebrafish
Source: Front Mol Neurosci. 2017 Apr 10;10:95. doi: 10.3389/fnmol.2017.00095 (PMC5385350; doi:10.3389/fnmol.2017.00095)

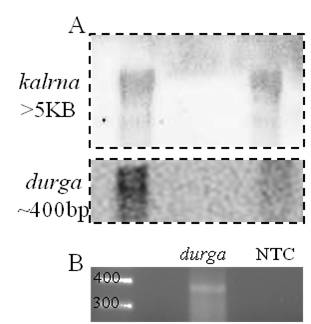

Supplement: FIGURE S1 — Northern blot representing transcript corresponding kalrna and lncRNA durga. Northern blotting experiment shows the presence of transcripts corresponding kalrna and durga above 5 KB and ~400 bp respectively (A). Polymerase chain reaction (PCR) detection of durga transcript using anchored oligodT reverse primer for cDNA synthesis and amplified using durga forward primer (B). [file Image_1.jpeg]

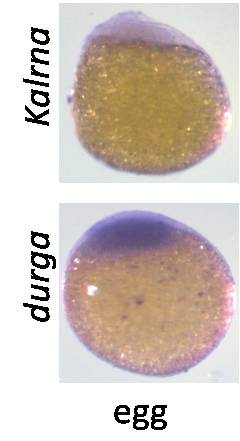

Supplement: FIGURE S2 — In situ hybridization of kalrna and durga in the zebrafish egg. In situ hybridization shows kalrna and durga transcript expression in zebrafish egg. [file Image_2.jpeg]
